# Supplementary material for: Large language models can extract metadata for annotation of human neuroimaging publications
Source: Front Neuroinform. 2025 Aug 20;19:1609077. doi: 10.3389/fninf.2025.1609077 (PMC12405296; doi:10.3389/fninf.2025.1609077)
Supplement: Supplementary file 1 [file Data_Sheet_1.pdf]

Supplement to:

Large Language Models Can Extract Metadata for Annotation of Human Neuroimaging Publications

Turner, et al.

Statistical Analysis

We present our results in the text of the main paper without detailed statistical analysis. This follows the practice in other contexts for the comparison of LLM performance (see, for instance, any LLM leaderboard such as LMArena as well as many other LLM performance comparison papers). As our claim was that LLM and human performance are *comparable*, and in every case that we present the LLM results were *better than or exactly equal to* the human results, this claim did not require a formal statistical analysis.

At the request of a reviewer, we provide a statistical analysis here. Note that our human data was often collected under different instructions than used for the LLM, and therefore the results presented in the paper are sometimes incommensurate as collected. Therefore, the analysis here will have to differ somewhat from the presentation in the paper. We will highlight these changes in the discussion below.

Statistical tests were done with the Python libraries `statsmodels` and `mlxtend`, and the code to compute them is available in the GitHub repository listed in the main paper.

Task 1: General Imaging Type

In this task, for the main analysis, we have two systems (human and LLM) annotating the publications. Both systems achieve identical accuracies (97.3%). Therefore, no statistical testing is required as the two-sided p-value is equal to 1, formally.

Task 2: Structural MRI Parameters

This task had a complicated structure as the annotations were added in phases by different annotators and the LLM was tasked with a more detailed parameter extraction. Note that throughout the graduate student errors are true errors while the LLM "errors" are mismatches, some of which are, in fact, correct. See the complete discussion of LLM mismatches in the main paper.

We only compare the initial, worst case, performance of the LLM against the human annotators as all of the revised accuracies in the discussion of this task in the paper prefer the LLM to humans by a wider margin.

Graduate Student Annotations

The graduate student annotators had a coarser collection requirement with 396 annotation positions. Here we map the LLM responses into this coarser frame for comparison, so in many cases *multiple* LLM mismatches map to a single error in this system.

For instance, the human annotators reported voxel size as a string and only complete correctness was counted as correct. The LLM reported each of the 3 dimensions as a separate number and these were counted independently. So, here the LLM making 1, 2, or 3 errors in a paper's voxel size would become a single error. Note that this goes both ways, because a single dimension being wrong (but the other two being right) would still be counted as a single error in this analysis, but the two correct dimensions would not be accounted for in the denominator. This also affects FOV and matrix parameters (see main paper for details).

Having done this mapping, we find the following error structure presented in a 2x2 table:

Table S1. 2x2 table for task 2 graduate student annotations.

		LLM	
		Correct	Incorrect
Human	Correct	360	13
	Incorrect	17	6

Note that the sum of all four cells is 396, the total of the annotation positions. Using McNemar's exact test we find a $p = 0.585$. Thus, there is not a significant difference between the performance of the graduate students and the LLM.

Additional Annotations

The additional annotations were added in a different phase. The corresponding 2x2 table for this case is:

Table S2. 2x2 table for task 2 additional annotations.

		LLM	
		Correct	Incorrect
Human	Correct	124	1
	Incorrect	7	0

Which results in a McNemar's exact test p = 0.070 and again the difference between the two systems is not significant. Note that we should not over interpret this "marginal" p-value, but if we did it would favor the LLM not the human annotator.

Task 3: Experimental Group Information

For this task, we did not directly compare human performance with the LLM on the same data. Against the gold standard data we used for testing, the LLM gave 11 errors (details in the paper). *Formally*, this produces the following 2x2 table:

Table S3. 2x2 table for task 3 experimental group information against gold standard.

		LLM	
		Correct	Incorrect
Gold Standard	Correct	109	11
	Incorrect	0	0

Which provides a McNemar' exact test p = 0.00098. However, this degenerate table simply demonstrates that we can distinguish a perfect result from one with 11 errors and is not relevant as it is not a comparison with human performance data.

We also made a comparison with human performance on an independent *convenience sample* of publications. Due to the use of different publications, there is no way to use a test like McNemar's for this case. We reported the performance in table 4 of the main paper. There we reported the LLM accuracy of 90.8% against a human accuracy of 83.3%, which analyzes the annotation positions separately.

It may appear that we can simply do a standard two-sample z-test of proportions but this assumes that the proportions are based on fully independent sampling which is not the case here as the papers analyzed had multiple annotation positions per paper and this leads to a

clear dependency *within* papers. Additionally, the human data was estimated using a conservative rule which treated each paper annotated as having a maximum of one error per paper. (See the main text for details.) To avoid this issue, we instead compare the "publication fraction correct," which is a coarser measure, but which meets the requirements for the two-sample z-test for proportions and additionally does justice to the conservative scoring procedure used for the human annotators.

Comparing the LLM publication fraction correct of 0.867 with a human fraction correct of 0.667. This yields a $z = 1.91$ with a $p = 0.056$. This difference does not meet the significance criterion, and therefore we conclude that there is no evidence that the performance differs between humans and LLMs for this case. Our comment above about over interpreting a marginal p-value applies here as well.

Statistical Conclusions

Our claim was that the LLM's performance is comparable to human annotation performance. In every case, LLM accuracy exactly matches or exceeds human accuracy. Despite reframing the analysis here, there are no results that change that conclusion.
